# Identification of Key Genes in Lung Adenocarcinoma and Establishment of Prognostic Mode

**DOI:** 10.3389/fmolb.2020.561456

**Published:** 2020-10-27

**Authors:** Zhou Jiawei, Mu Min, Xing Yingru, Zhang Xin, Li Danting, Liu Yafeng, Xie Jun, Hu Wangfa, Zhang Lijun, Wu Jing, Hu Dong

**Affiliations:** ^1^School of Medicine, Anhui University of Science and Technology, Huainan, China; ^2^Key Laboratory of Industrial Dust Prevention and Control and Occupational Safety and Health, Ministry of Education, Anhui University of Science and Technology, Huainan, China; ^3^Affiliated Cancer Hospital, Anhui University of Science and Technology, Huainan, China

**Keywords:** lung adenocarcinoma, bioinformatics, prognosis, predictor, data mining

## Abstract

**Background:**

The development of human tumors is associated with the abnormal expression of various functional genes, and a massive tumor-based database needs to be deeply mined. Based on a multigene prediction model, access to urgent prognosis of patients has become possible.

**Materials and Methods:**

We selected three RNA expression profiles (GSE32863, GSE10072, and GSE43458) from the lung adenocarcinoma (LUAD) database of the Gene Expression Omnibus (GEO) and analyzed the differentially expressed genes (DEGs) between tumor and normal tissue using GEO2R program. After that, we analyzed the transcriptome data of 479 LUAD samples (54 normal tissue samples and 425 cancer tissue samples) and their clinical follow-up data from the (TCGA) database. Kaplan–Meier (KM) curve and receiver operating characteristic (ROC) were used to assess the prediction model. Multivariate Cox analysis was used to identify independent predictors. TCGA pancreatic adenocarcinoma datasets were used to establish a nomogram model.

**Results:**

We found 98 significantly prognosis-related genes using KM and COX analysis, among which six genes were found to be the DEGs in GEO. Using multivariate analysis, it was found that a single gene could not be used as an independent predictor of prognosis. However, the risk score calculated by weighting these six genes could serve as an independent prognosis predictor. COX analysis performed with multiple covariates such as age, gender, tumor stage, and TNM typing showed that risk score could still be utilized as an independent risk factor for patient survival rate (*p* = 0.013) and had an applicable reliability (area under the curve, AUC = 0.665). By combining risk score and various clinical features, the nomogram model was constructed, which had been proven to have high consistency for the prediction of 3- and 5-year survival rate (concordance = 0.751) and high accuracy as tested by ROC (AUC = 0.71;AUC = 0.708).

**Conclusion:**

We proposed a method to predict the prognosis of LUAD by weighting multiple genes and constructed a nomogram model suitable for the prognostic evaluation of LUAD, which could provide a new tool for the identification of therapeutic targets and the efficacy evaluation of LUAD.

## Introduction

At present, pulmonary tumors have become the malignant tumor with the highest morbidity and mortality in China (Siegel et al., 2018). Among them, non-small cell lung cancer (NSCLC) accounts for more than 85% of newly diagnosed cases, and lung squamous cell carcinoma (LUSC) and lung adenocarcinoma (LUAD) are the two major pathological types of NSCLC (Lawrence et al., 2013). Recently, LUAD had surpassed LUSC in increasing morbidity and became the most common clinicopathologic type (Bray et al., 2018). Epidemiological studies showed that LUAD was associated with risk factors such as smoking, drinking, staying up late, and metabolic disorders. Although various treatment strategies (*e*.*g*., surgery, chemotherapy, radiotherapy, and biological agents) have made progress, the effective diagnosis and prognosis prediction of LUAD patients are still huge challenges in clinical practice (Park et al., 2019).

Tumor occurrence is a complicated process along with genetic gene changes at the same time, and these altered genes usually show abnormal expression patterns, thus having clinical significance for the diagnosis and prognosis of cancer (Tang et al., 2018). Presently, some molecules have been considered as markers for the diagnosis and prognosis of LUAD. For example, carcinoembryonic antigen (CEA) is one of the most specific carcinoembryonic proteins and one of the most widely used tumor markers. In fact, 40–80% of lung cancer patients may have elevated serum CEA. The degree of serum CEA increase is related to the extent of cancer focus, and its dynamic changes can reflect the patient’s response to treatment and prognosis. Patients with a progressive increase in the measured value tend to have a poor prognosis, while patients with firstly decreased and then increased values are mostly experiencing signs of tumor recurrence (Zheng et al., 2020). Glutathione-*S*-transferase-π (GST-π) is significantly increased in patients with NSCLC. The increase of GST-π indicates that the tumor is insensitive to radiotherapy and chemotherapy and has a poor prognosis (Zhao et al., 2018). Similarly, p53 gene is mutated in about 60% of patients with NSCLC, and its mutation often occurs in the early stage of tumor occurrence, so if specimens can be obtained in time, it will be helpful for early diagnosis (Wu et al., 2020). However, due to the limitations of sensitivity and specificity, the existing biomarkers are not suitable for all LUAD cases. Therefore, the screening and the identification of new functional genes are urgent to understand the pathogenesis of tumors and improve the accuracy of diagnosis and prognosis of LUAD.

Gene expression profile array could be used to identify differentially expressed genes (DEGs) between tumor samples and normal samples (Dong et al., 2020), and some key genes can be identified by bioinformatics mining (Xiao et al., 2020). However, integrating the contributions of several key genes to improve the accuracy and the sensitivity of tumor prognosis judgment has always been a difficult problem in its clinical application. Therefore, this study used bioinformatics analysis to screen six prognosis-related DEGs from the Gene Expression Omnibus (GEO) and The Cancer Genome Atlas (TCGA) databases and used risk score, which represented the predictive power of multiple genes, as an independent predictor of tumor prognosis by calculating risk score values and constructing a nomogram model, showing higher accuracy.

## Materials and Methods

### Data Collection

Gene Expression Omnibus is a gene expression data warehouse that collects gene expression data in any animal species (Barrett et al., 2013). In this study, we first downloaded three RNA expression profiles (GSE32863, GSE10072, and GSE43458) of LUAD from the GEO public database^1^. The selection criteria for the expression profile are as follows: (1) the test samples are tissues, (2) all tissues are diagnosed as LUAD tissues and normal tissues, (3) the gene expression profile is mRNA, (4) samples were collected from the same ethnic group, (5) the probes can be converted into the corresponding gene symbols, and (6) complete information analysis. The array data of GSE32863 includes 58 LUAD tumor tissues and matched adjacent normal tissue cases (Selamat et al., 2012), GSE10072 includes 54 LUAD tumor tissues and 54 normal tissue cases (Landi et al., 2008), and GSE43458 includes 80 tumor tissues and 30 normal tissue cases (Kabbout et al., 2013).

### Data Processing

The differences between LUAD and normal samples were analyzed by GEO2R^2^, a built-in online tool of GEO. The significance of DEGs was evaluated by the adjusted *P* value and | log fold change| (| log FC|), and the adjusted *P* < 0.05 and | log FC| > 1 were used as screening criteria.

### Construction of Prognostic-Related Gene Screening and Prediction Model

In the TCGA^3^ database, we downloaded 479 LUAD transcriptome data, including 54 normal tissue samples and 425 cancer tissue samples as well as clinical follow-up data (Gao et al., 2013). According to R language analysis results of gene chip and transcriptome data, a total of 8,108 abnormally expressed genes were obtained, from which we used R language to screen 98 prognostic genes significantly related to prognosis, analyzed the genes screened in GEO and TCGA, and finally got six abnormal expression genes significantly related to the prognosis of LUAD. GEPIA was used to verify the expression and prognostic potential of the six genes in ten different types of tumors. Subsequently, we carried out univariate and multivariate prognosis analyses on the selected genes, constructed a Cox proportional hazard regression model to fit the multi-gene prediction model, and then conducted a survival analysis. The prediction efficiency evaluation of the model adopted the area under curve (AUC) of the time-dependent receiver operating characteristic curve (ROC), and then we drew the risk curve and evaluated the gene model as an independent predictor and the related clinical feature analysis. Finally, predictive risk models of the six genes (VIPR1, FCN3, CA4, CRTAC1, CYP4B1, and NEDD9) were obtained.

### Gene Risk Score and Clinical Feature Prognosis Model

We calculated the individual prognostic risk score of six genes and the mixed risk score of six genes, combined with clinical data, and then constructed a gene risk score and clinical feature prognosis model. Calibration and ROC were used to evaluate the constructed model.

### Statistical Analysis

All statistical analyses were performed using R v. 3.4.3 and GraphPad Prism 7.0 (GraphPad Software, Inc., San Diego, CA, United States). The relationship between markers was assessed by Pearson’s correlation and regression. For comparisons, two-tailed Student’s *t*-test and Welch’s *t*-test were applied, as appropriate. Univariate- and multivariate Cox regression analyses were performed to evaluate survival. Unless otherwise stipulated, *P* < 0.05 was considered statistically significant.

## Results

### Identification of DEGs of LUAD in GEO

**FIGURE 1:**
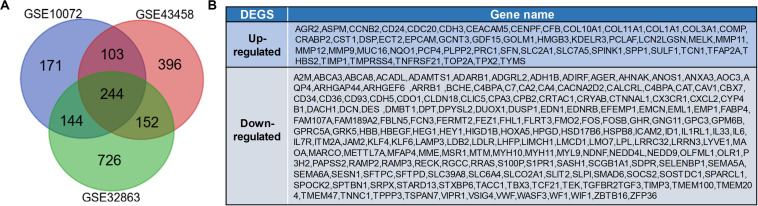
Identification of differentially expressed genes (DEGs) in lung adenocarcinoma. **(A)** Venn diagram of DEGs in GSE10072, GSE43458, and GSE32863. The DEGs with *P*-value <0.05 and a fold change >1 were selected. **(B)** Expression of 244 DEGs in tumor tissues.

According to the analysis of GEO2R, DEGs (1,266 in GSE32863, 662 in GSE10072, and 895 in GSE43458) were identified. Among these DEGs, a total of 244 abnormally expressed genes which have abnormal expression were extracted from three datasets (Figure 1A), including 51 up-regulated genes and 193 down-regulated genes (Figure 1B).

### Screening of Genes Related to the Prognosis of LUAD

**FIGURE 2:**
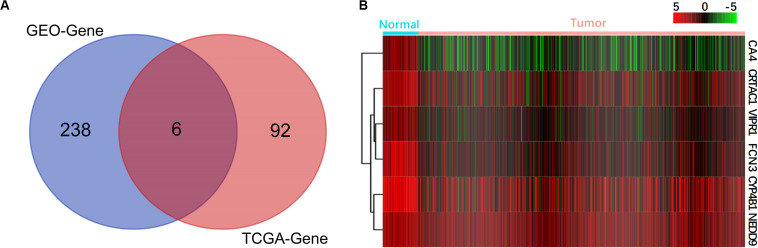
Screening of genes related to the prognosis of lung adenocarcinoma. **(A)** Venn diagram of prognosis-related genes. **(B)** The expression heat maps of six selected prognostic genes are shown, with red indicating high expression and green indicating low expression.

To confirm the correlation between DEGs and prognosis, we downloaded the original transcriptome data of 479 LUAD samples from the TCGA database, which included 54 normal tissue samples, 425 cancer tissue samples, and clinical follow-up data. We screened 8,108 DEGs by using R language with *P* < 0.05 and |log FC| > 1 as the standard. Based on clinical prognostic data, KM and COX algorithm had screened 98 significantly prognosis-related genes from 8,108 genes, and six of them were found to be the same as the DEGs from GEO (Figure 2A), namely, VIPR1, FCN3, CA4, CRTAC1, CYP4B1, and NEDD9. Consistent with the results of the GEO dataset (Figure 1B), these six genes were also down-expressed in the TCGA dataset (Figure 2B). The expression and prognosis of 6 genes were verified by 10 kinds of cancer data in GEPIA database. It was found that only 6 genes in LUAD had differential expression (Supplementary Figure S1) and correlation with prognosis (Supplementary Figure S2).

### Prognostic Analysis of LUAD by Univariate and Multivariate Analyses

**FIGURE 3:**
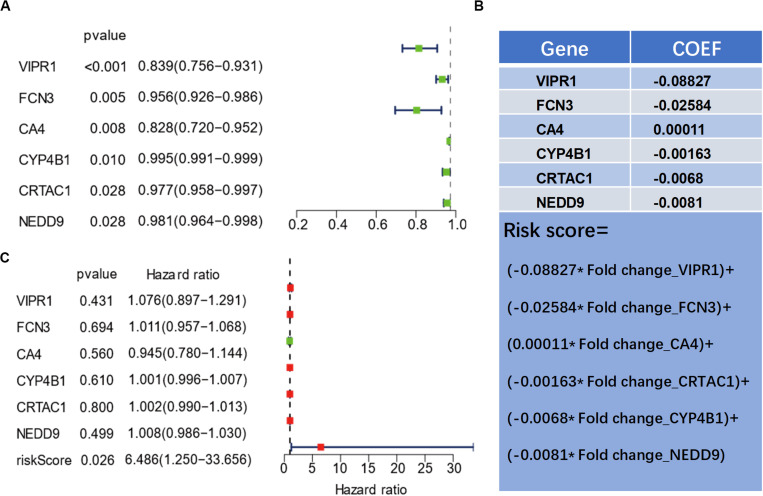
Univariate and multivariate prognostic analyses of genes related to the prognosis of lung adenocarcinoma. **(A)** Dendrogram of six genes by univariate prognostic analysis. **(B)** The calculation formula of the comprehensive risk score of the six genes. **(C)** Results of the risk score by multivariate prognostic analysis.

In order to further examine the correlation between the six genes and the prognosis of patients, we selected 425 LUAD samples from the TCGA database and performed univariate and multivariate analyses on the prognosis of the selected genes (Figures 3A,B). Using the log-rank method, a univariate analysis revealed that the six genes were all significantly related to the prognosis of the patients (*P* < 0.05) (Figure 3A). To clarify the correlation among the expression levels of the six genes and whether they could be used as independent predictors of prognosis, we used the COX method for multivariate analysis and found that the expression level of the six genes is correlated. Meanwhile, we also found that a single gene could not serve as an independent predictor of prognosis (Figure 3A). To evaluate the overall efficacy of the six genes as an assessment of prognosis, we integrated the six genes and calculated a comprehensive risk score (Figure 3C) that was later utilized as a single variate. After COX analysis, we found that the comprehensive risk score of the six genes was significantly related to the prognosis (*P* = 0.026), suggesting that the comprehensive risk score of the six genes could be used as an independent predictor of prognosis (Figure 3B).

### Evaluation of the Prognosis Model

**FIGURE 4:**
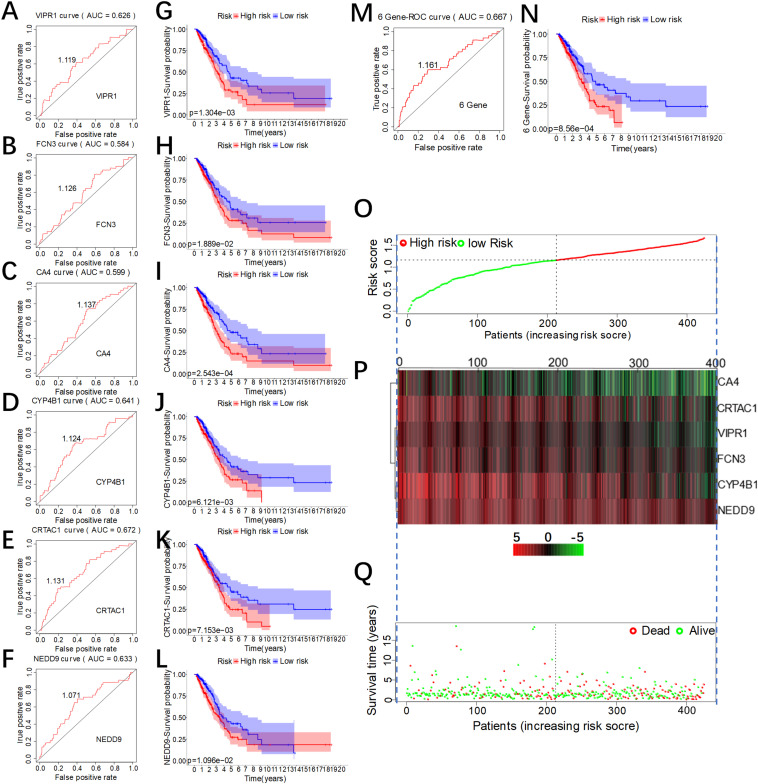
Evaluation of the predictive effect of the comprehensive risk score of the six genes. **(A–F)** The receiver operating characteristic (ROC) curve of the individual risk scores of the six genes. **(G–H)** Kaplan–Meier (KM) survival curves of lung adenocarcinoma (LUAD) patients with low or high individual risk score of the six genes. **(N)** KM survival curve of LUAD patients with low or high comprehensive risk score of the six genes. **(M)** The ROC curve of the comprehensive risk score of the six genes. **(O)** Risk score of patients, with red indicating high risk and green indicating low risk. **(P)** Expression heat map of the six genes in the high-risk group and the low-risk group (green for low expression and red for high expression). **(Q)** Distribution of the survival status of patients in the high-risk group and the low-risk group (red for death and green for survival).

According to the formula in Figure 3B, we separately calculated the risk score values of six genes and analyzed the reliability of the overall survival rate predicted by the individual risk score of the six genes using ROC curves (Figures 4A–F). The results found that the area under the ROC curve of CRTCA1 (AUC = 0.672) was the largest (Figure 4E), while that of FCN3 (AUC = 0.584) was the smallest (Figure 4B). NEDD9 has a paralogue CASS4 with similar function. Similarly, we found that CASS4 was also be associated with OS prediction (Supplementary Figures S3A–E) and low expression in LUAD (Supplementary Figures S3F–G). Using the optimal threshold of the ROC curve as a cutoff value, we divided 425 patients into two groups. The group with a value greater than the threshold value was recognized as the high-risk group, and accordingly the group with equal or lower values was the low-risk group. The difference in overall survival time of the six genes was calculated by the Kaplan–Meier survival curve (Figures 4G–L). Results showed that there were differences in the overall survival time between patients with high level and low level of FCN3 (*P* = 2.543E-04), but the ROC validation was poor (AUC = 0.548). Subsequently, we used the ROC curve to analyze the reliability of the overall survival rate predicted by the comprehensive risk score of the six genes. The area under the ROC curve was 0.667 (Figure 4M), indicating that the model had a good predictive ability. Additionally, the optimal threshold of the ROC curve was 1.316 (Figure 4M), according to which 425 patients were divided into two groups, the high-risk group (*n* = 213) with a value greater than 1.316 and the low-risk group (*n* = 212) with a value less than or equal to 1.316. We found that there was a difference in the overall survival time between the two groups. The overall survival time of patients in the high-risk group was significantly lower than that in the low-risk group, and the result of the log rank test was *P* = 8.56e-04 (Figure 4N). In addition, we also visualized the risk score in the high-risk group and the low-risk group (Figure 4O). It was found that, with the rise of risk score, the expression level of the six genes decreased (Figure 4P), while the survival rate of patients decreased (Figure 4Q), which confirmed that the comprehensive risk score did have a predictive effect of survival and prognosis.

### Evaluation of the Six-Gene Model as an Independent Predictor

**FIGURE 5:**
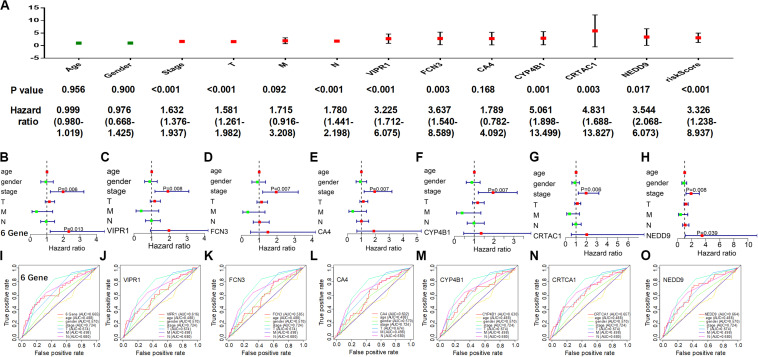
Evaluation of the six-gene model as an independent predictor. Individual and comprehensive risk scores were involved in a multivariate analysis with patient characteristics. **(A)** Univariate analysis. **(B–H)** Multivariate analysis. **(I–O)** Receiver operating characteristic curve.

As the individual characteristics of different patients could affect their survival rate, we included gender, age, tumor stage, and tumor classification (T,M,N) into our analysis and established a multivariate Cox proportional risk model after calculating the individual and the comprehensive risk scores of six genes. In the univariate analysis (Figure 5A), clinical characteristics (stage, T, N), VIPR1, and six-gene comprehensive risk score were all significantly associated with prognosis (*P* log-rank test <0.01).

In the multivariate analysis (Figures 5B–H), only the individual prognostic risk score of NEDD9 was significantly correlated with prognosis (*P* = 0.039), but the tumor stage was always significantly correlated. At the same time, the six-gene comprehensive risk score showed a stronger prognostic correlation (*P* = 0.013), suggesting that the six-gene comprehensive risk score could be used as an independent predictor. The ROC curve was used to evaluate the reliability of the prognosis predicted by the individual or the comprehensive risk score of six genes (Figures 5I–O), and it was found that the reliability of the prediction model of the six-gene comprehensive risk score (AUC = 0.665) was higher than that with the individual risk score. Therefore, tumor stage played an important role in tumor prognosis, and our constructed multi-genic prognostic model similarly exhibited a good ability to predict the prognosis of LUAD patients.

### Correlation Between Prognosis-Related Genes and the Clinical Features of LUAD

**FIGURE 6:**
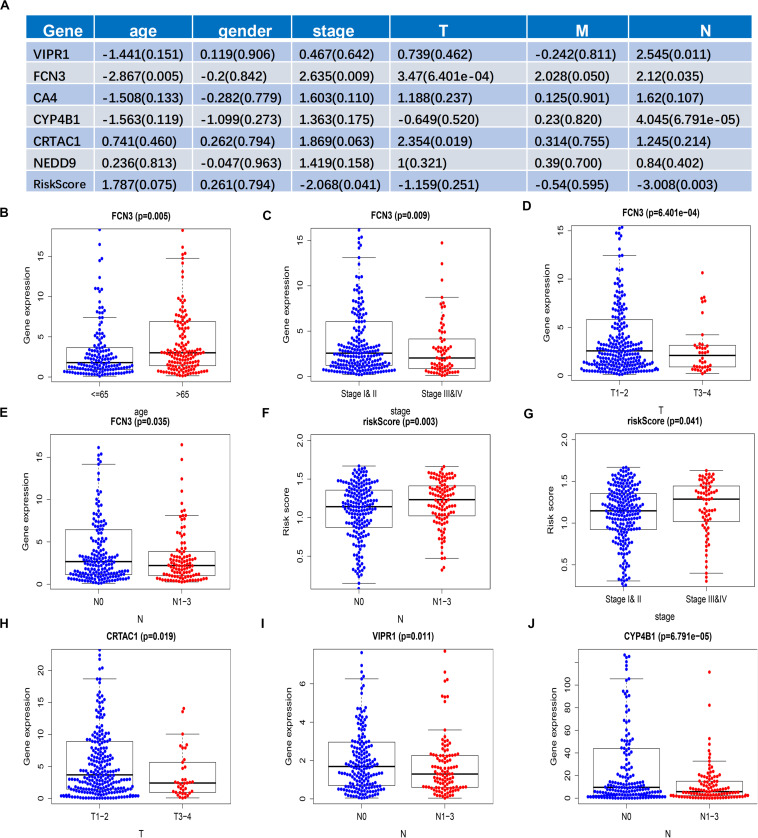
Correlation analysis of the individual expression and the comprehensive risk values of six genes with clinical features. **(A)** Results of the correlation analysis of the expression alone of the six genes and of the comprehensive risk value of the six genes with clinical features. Data are shown as *R* value (*p* value). **(B–J)** Box plot of the expression and the comprehensive risk score of the six genes of patients grouped with clinical characteristics.

To further verify the correlation between the six genes that we screened and the patients’ gender, age, stage, and TNM, we selected the transcriptome data of LUAD in TCGA and analyzed the correlation between the expression levels of the six genes and the clinical characteristics of the patients: gender was divided into two groups: male and female; age was divided into two groups: >65 years old and ≤65 years old; tumor stage was divided into two groups, stage I and II and stage III and IV; T was divided into two groups: T1–2 and T3–4; M was divided into two groups: M0 and M1; and N was divided into two groups: N0 and N1–3. The R (bee swarm) package was used to calculate the correlation between each gene and clinical characteristics, and the screening condition was *p* < 0.05 (Figure 6A). FCN3 and age (Figure 6B), tumor stage (Figure 6C), T, and N were all correlated (Figures 6E,F), but not with T (Figure 6D), of which T (*P* = 6.401E-04) was most significantly correlated with FCN3, suggesting that FCN3 might play an important role in the size and the metastasis of primary tumor. Risk score calculated by six genes mixing was correlated with N and stage (Figures 6F,G), and the risk value and the patient’s mortality rate increased with the rise of N and stage. CRTAC1, VIPR1, and CYP4B1 were respectively related to T stage (Figure 6H), N stage (Figure 6I), and N stage (Figure 6J). In addition, with the rise of stage, the expression level of three genes decreased in stage, which could be contributed to the fact that these three genes were all low-risk genes with a decreased expression, reduced survival of patients, and poor prognosis.

### Genetic Risk Score and Establishment and Evaluation of Prognosis Model of Clinical Features

**FIGURE 7:**
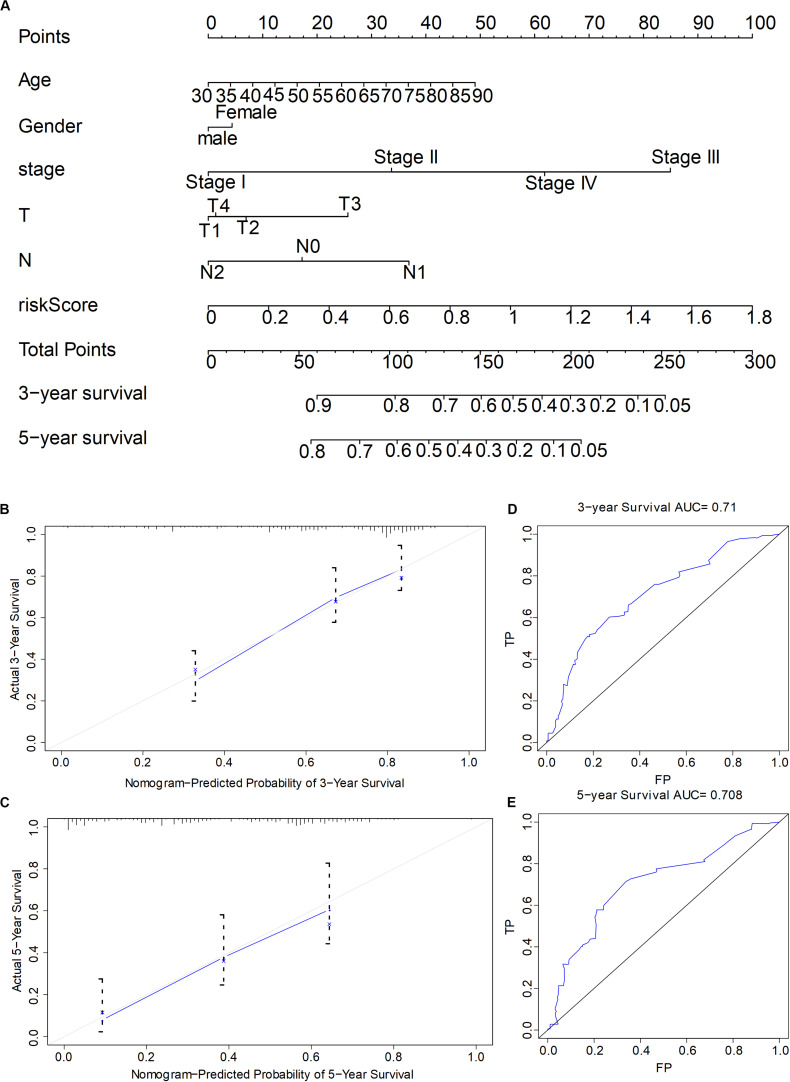
The establishment and the evaluation of the prognostic model combining the comprehensive risk score of six genes with clinical features. **(A)** Nomogram of the prognostic model. **(B,C)** Calibration tested the accuracy of the constructed model to predict the 3- and 5-year survival status, respectively. **(D,E)** Receiver operating characteristic tested the accuracy of the constructed model to predict the 3- and 5-year survival status, respectively.

To quantify the prognosis evaluation of LUAD patients, we selected six genes according to parameters such as risk score, gender, age, stage, and TNM, and then we constructed the nomogram model with 3-year survival rate and 5-year survival as evaluation indicators. The clinical data of LUAD in TCGA were chosen and grouped by the following order: gender was divided into male and female; tumor stage was divided into stage I, stage II, stage III, and stage IV; T was divided into T1, T2, T3, and T4; N was divided into N0, N1, N2, and comprehensive risk score. The correlation between each factor and survival time was calculated by R language program, respectively, and the nomogram was drawn later (Figure 7A). Calibration was used to calculate the concordance of the model (concordance = 0.751), and the 3- and 5-year survival curves coincided, indicating the reliability of the model (Figure 7B). Subsequently, ROC curve also verified the accuracy of the model, which displayed that the AUCs of 3- and 5-year survival states were 0.71 and 0.708, respectively, suggesting that the model had a high accuracy.

## Discussion

Global statistics about cancer show that lung cancer is the most common malignancy and the leading cause of cancer-related deaths, accounting for 11.6% of all malignancies and 18.4% of cancer-related deaths. LUAD is the most common subtype of malignant lung cancer, and its incidence is rapidly increasing. Air pollution and smoking are two important risk factors for LUAD (Li et al., 2019), which can cause multiple genetic mutations in lung cancer patients (Guo et al., 2019). However, due to the high heterogeneity among tumor cells, there are huge differences in the signaling pathways and key gene expression that regulate tumor growth, migration, transformation, and drug resistance. Besides that, there are complementary compensatory effects among the tumor-promoting mechanisms (Jing et al., 2019a,b), which also pose great challenges to tumor diagnosis, treatment, and prognosis. So far, no ideal tumor marker for the diagnosis and the prognosis evaluation of LUAD has been found out. Even common clinical biomarkers such as TTF-1, NAPSA, and CEA need to be combined to diagnose LUAD effectively, but they are not proto-oncogenes, and these indicators are not effective in predicting prognosis. It has been reported that microarray technology can detect the changes of tumor gene expression profile with high throughput. It is an effective method for screening biomarkers for the early diagnosis and prognosis evaluation of LUAD (Eriksson and Sjödahl, 2019; Jing et al., 2019a,b). In this study, we screened DEGs of LUAD by RNA expression profile analysis, then screened out six key genes by prognostic correlation analysis, and calculated the comprehensive risk score of the six genes by assignment. Through the validation model, we confirmed that the index could be used as an independent predictor of survival rate of LUAD patients and constructed the nomogram model to provide the quantity indicators of prognosis risk assessment for LUAD patients.

Based on different data sources and design ideas, bioinformatics analysis usually uses different database information to verify each other for improving the accuracy and the applicability of the analysis results (Hu et al., 2019). First, we downloaded three mRNA microarray datasets from the GEO database and analyzed them. A total of 51 up-regulated genes and 193 down-regulated genes were identified (Figure 1). We used 479 original transcriptome data of LUAD in the TCGA database to verify and screen out 8,108 DEGs. To confirm the correlation between DEGs and the prognosis of LUAD patients, we used COX and KM algorithm to screen out 98 differentially expressed prognostic genes that were significantly related to the prognosis. After analyzing the genes screened from GEO and TCGA comprehensively, we finally obtained six key genes (VIPR1, FCN3, CA4, CRTAC1, CYP4B1, and NEDD9) (Figure 2).

Vasoactive intestinal peptide receptor-1 (VIPR1) has a significant growth effect on many common tumors, and it is lowly expressed in human LUAD tissues and lung cancer cell line H1299. When overexpressed, it is found to significantly inhibit the growth, migration, and invasion of lung cancer cells (Zhao et al., 2019). This study showed that LUAD, KIRC and LIHC patients with low VIPR1 expression had poor prognosis (Supplementary Figure S2A), which was consistent with the findings in liver cancer and cervical cancer (Kim et al., 2013; Lu et al., 2019). Fibronectin 3 (FCN3), encoded by the FCN3 gene, is a recognition molecule in the lectin pathway of the complement system (Munthe-Fog et al., 2009). It is closely related to immune function and is highly expressed in normal human lung tissues but lowly expressed in lung cancer tissues (Hummelshoj et al., 2008). LUAD and LIHC patients with low expression of FCN3 have poor prognosis, which is consistent with the findings in liver cancer and esophageal cancer (Yu et al., 2017; Li et al., 2019). Carbonic anhydrase (CA4) is zinc metalloenzyme that catalyzes the reversible hydration of carbon dioxide. It participates in a variety of biological processes, including respiration, calcification, acid–base balance, bone resorption, and formation of aqueous humor, cerebrospinal fluid, saliva, and gastric acid. It is down-expressed in human LUAD tissues (Yu et al., 2020) and promotes the proliferation of cancer cells (Chen et al., 2017). The prognosis of LUAD, KIRC and LGG patients with low expression of CA4 is poor, which is consistent with the findings in colorectal cancer and renal cell carcinoma (Takenawa et al., 1998; Liu et al., 2020). Cartilage acidic protein 1 (CRTAC1) is a glycosylated extracellular matrix protein, which exists in the interregional matrix of cartilage in the particularly deep region and participates in cell-to-cell or cell-to-matrix interactions. It is highly expressed in normal human lung tissues (Ballard et al., 2010) and poorly expressed in LUAD tissues. Patients with LUAD, BLCA and LGG with low expression of CRTAC1 have poor prognosis, which is consistent with that found in glioma (Xiao et al., 2020). Cytochrome P450 4B1 (CYP4B1) is a tissue-specific detoxification-related monooxygenase and is down-expressed in LUAD, but its role in tumorigenesis is still unclear (Lim et al., 2020; Ni and Sun, 2019). The prognosis of LUAD, CESC and KIRC patients with low expression of CYP4B1 is poor, which is consistent with the findings of urethral cancer and adrenal cancer (Genter et al., 2006; Murtha et al., 2017; Lin et al., 2019). Neural precursor cell expressed developmentally downregulated 9 (NEDD9) is a cytoskeletal protein molecule that is related to biological functions such as cell adhesion, migration, invasion, apoptosis, and cell cycle and promotes cancer metastasis (Samokhin et al., 2018). Animal experiments had shown that NEDD9, in combination with miR−363−3p, could inhibit NSCLC (Chang et al., 2020). CASS4 is a paralogue of NEDD9 gene, and LUAD patients with low expression of CASS4 have poor prognosis (Supplementary Figure S3). It has similar expression pattern and prognostic effect to NEDD9 (Singh et al., 2008; Kondo et al., 2012; Li et al., 2016), and is related to the prognosis of many cancer patients such as gastric cancer and glioma (Speranza et al., 2012; Shi et al., 2014). Consistent with previous studies, the expression profile data selected in this study showed that the six genes were down-expressed in LUAD tissues.

Compared with a single biomarker, the combination of multiple biomarkers will improve the accuracy and the reliability of tumor prognosis prediction. Therefore, we analyzed the correlation between the six genes and prognosis by using univariate Cox and found that VIPR1 had the strongest correlation with the prognosis of patients. However, based on multivariate analysis, we discovered that only the comprehensive risk score of the six genes was correlated with prognosis (Figure 3), which might be due to the synergistic expression between the six genes. Furthermore, ROC curve was used to verify that the predictive model of the comprehensive risk score of the six genes was more effective.

The individual and the clinical features of tumor patients are important factors affecting the prognosis. Therefore, gender, age, stage, and TNM of patients were included in our analysis, and the correlation among independent and comprehensive risk scores of the six genes and the clinical features and the prognosis was analyzed, respectively. It was found that tumor stage and comprehensive risk score were strongly linked to prognosis. ROC assessment showed that stage had the highest accuracy, and comprehensive risk score took the second place. Therefore, we again verified the important role of stage in the prediction of tumor prognosis (Bao et al., 2020) and found that the comprehensive risk score of the six genes could be used as an independent predictor of the prognosis of LUAD. In addition, the comprehensive risk score also had a significant correlation with the patient’s clinical features, which was positively correlated with stage and N. In other words, the higher the comprehensive risk score was, the worse the patients’ prognosis and the condition were. This also reminded us that when we combined the comprehensive risk score and stage to predict the prognosis of patients, more accurate results might be achieved.

Quantifying the contribution degree of prognostic factors could provide an important data support and a convenient means for clinical treatment and doctor–patient communication. We built a visual and quantitative prognosis model based on six-gene comprehensive risk score and clinical features (Figure 7), which were used to predict the 3- and 5-year survival status of patients. The predicted results had a high consistency (model concordance = 0.751), and the accuracy of the model was high. The AUC of the ROC curve of 3- and 5-year survival was 0.71 and 0.708, respectively. Therefore, our nomogram model could provide the specific quantitative values of contribution degree of different predictors and prognosis evaluation for the clinic.

In conclusion, this study provided a method to convert the expression levels of multiple genes into an independent predictor of the prognosis of LUAD and constructed a quantitative model for its predictive efficacy, which would have great potential application value in guiding the clinical treatment and the prognosis assessment of LUAD. However, the reliability and the scope of use of the comprehensive risk score and quantitative model of the six genes used in this study remained to be verified by larger clinical cohort studies.

## Data Availability Statement

All datasets presented in this study are included in the article/Supplementary Material.

## Author Contributions

HD and WJ contributed to the conception and design of and supervised the study. ZJ, MM, ZX, LD, LY, and ZL developed the methodology, analysis, and interpretation of data and wrote the manuscript. XY, XJ, and HW reviewed the manuscript. All the authors listed have approved the manuscript.

## Conflict of Interest

The authors declare that the research was conducted in the absence of any commercial or financial relationships that could be construed as a potential conflict of interest.

## Footnotes

1 http://www.ncbi.nlm.nih.gov-/geo

2 http://www.ncbi.nlm.nih.gov/geo/geo2r

3 http://www.oncomine.org

## Supplementary Material

Supplementary Figure 1 The expression of 6 gene. **(A–F)** VIPR1, FCN3, CA4, CRTAC1, CYP4B1 and NEDD9 in GEPIA expression box diagram in 10 types of cancer.

Supplementary Figure 2 The prognosis of 6 gene. **(A–F)** The prognostic curves of VIPR1, FCN3, CA4, CRTAC1, CYP4B1 and NEDD9 in 10 types of cancer in GEPIA.

Supplementary Figure 3 Evaluation CASS4 as an independent predictor. **(A–E)** CASS4 gene risk scoring effect and ROC curve. **(F–G)** The expression of cass4 in LUAD.
