# Supplementary figures and images for: Identification of Key Genes in Lung Adenocarcinoma and Establishment of Prognostic Mode
Source: Front Mol Biosci. 2020 Oct 27;7:561456. doi: 10.3389/fmolb.2020.561456 (PMC7653064; doi:10.3389/fmolb.2020.561456)

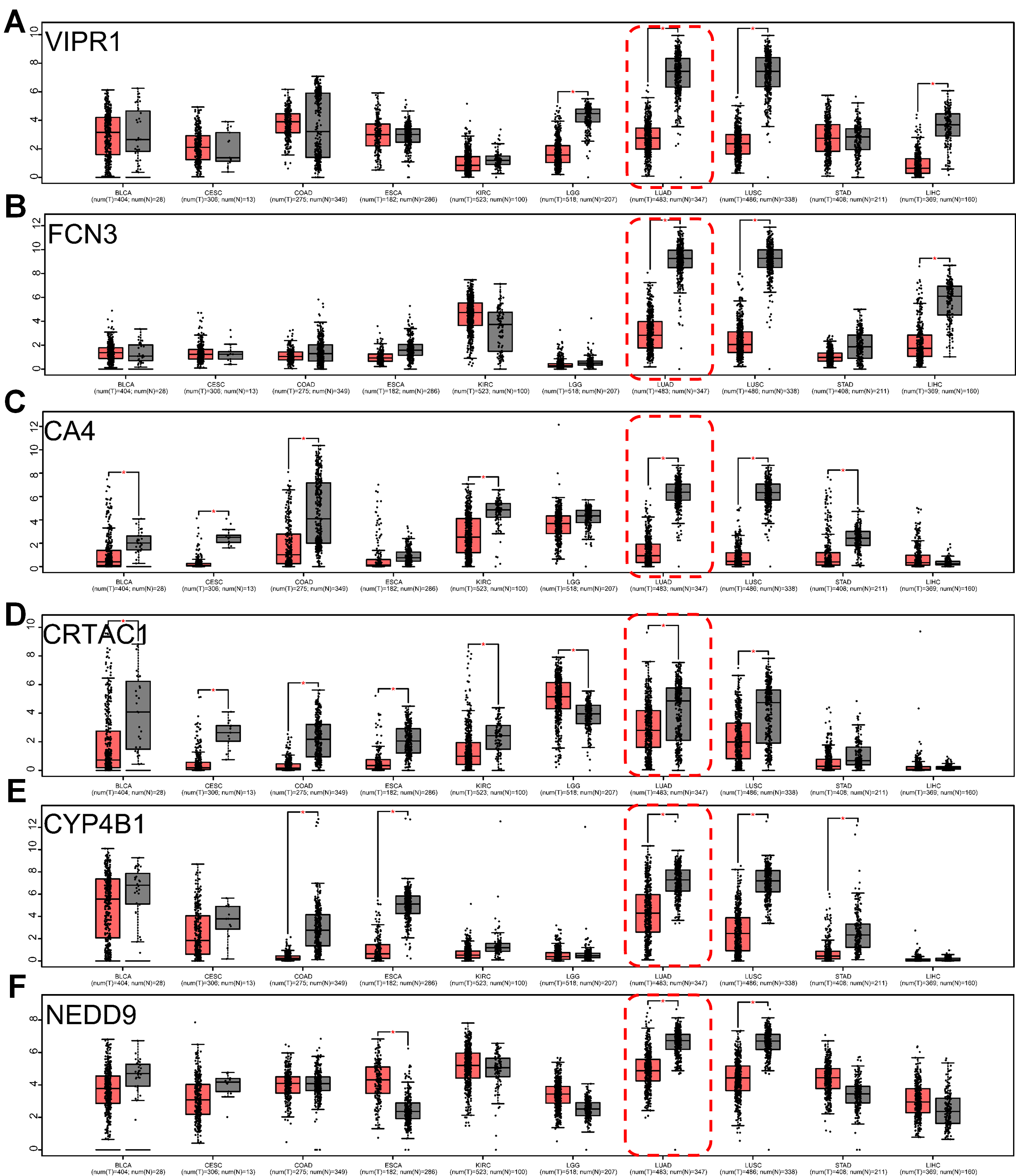

Supplement: Supplementary Figure 1 — The expression of 6 gene. (A–F) VIPR1, FCN3, CA4, CRTAC1, CYP4B1 and NEDD9 in GEPIA expression box diagram in 10 types of cancer. [file Image_1.tif]

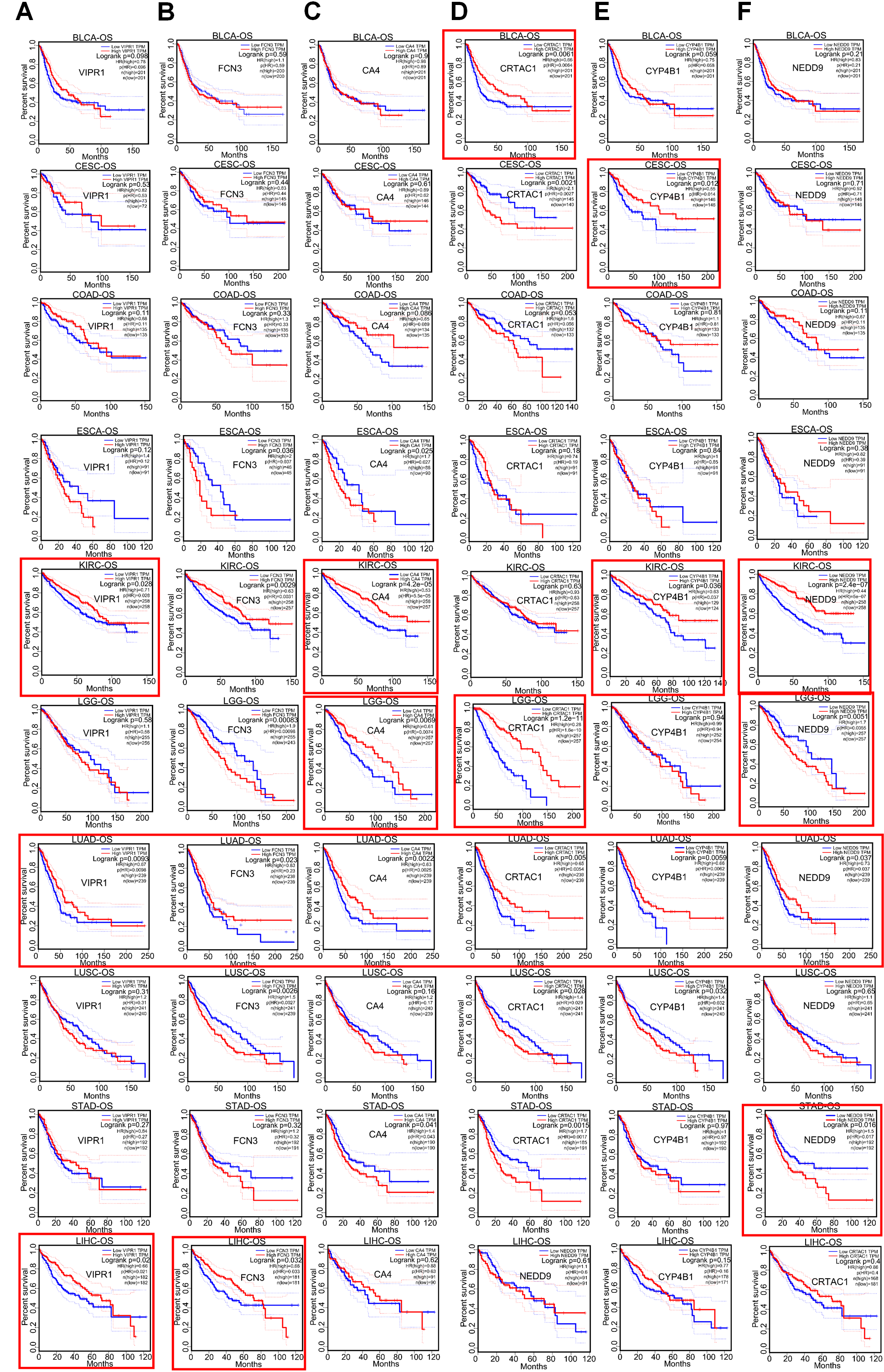

Supplement: Supplementary Figure 2 — The prognosis of 6 gene. (A–F) The prognostic curves of VIPR1, FCN3, CA4, CRTAC1, CYP4B1 and NEDD9 in 10 types of cancer in GEPIA. [file Image_2.tif]

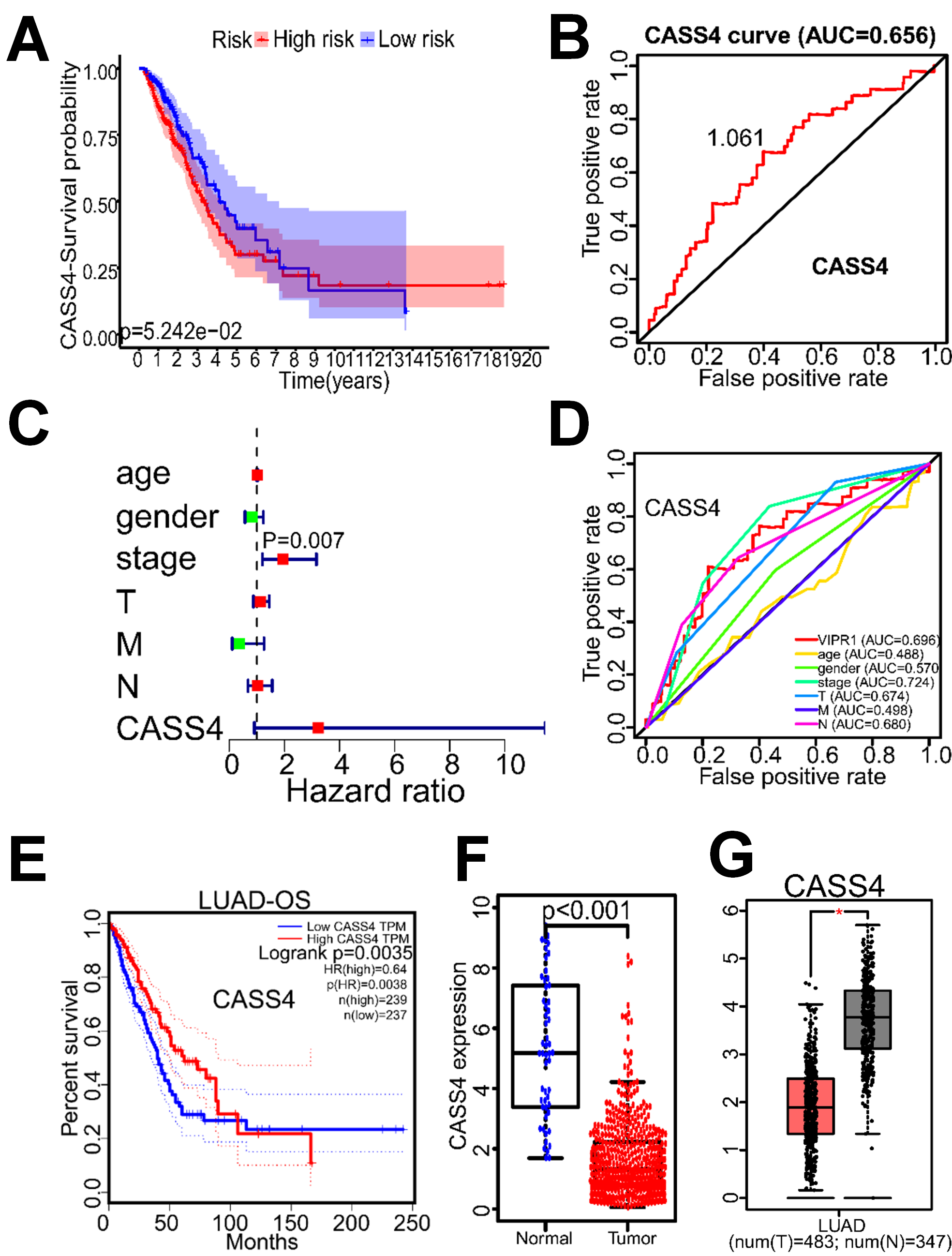

Supplement: Supplementary Figure 3 — Evaluation CASS4 as an independent predictor. (A–E) CASS4 gene risk scoring effect and ROC curve. (F–G) The expression of cass4 in LUAD. [file Image_3.tif]
